# Antifungal susceptibility and molecular characteristics of *Cryptococcus* spp. based on whole-genome sequencing in Zhejiang Province, China

**DOI:** 10.3389/fmicb.2022.991703

**Published:** 2022-11-17

**Authors:** Junli Zhang, Zhengan Wang, Yan Chen, Zhihui Zhou, Qing Yang, Ying Fu, Feng Zhao, Xi Li, Qiong Chen, Li Fang, Yan Jiang, Yunsong Yu

**Affiliations:** ^1^Department of Infectious Diseases, Sir Run Run Shaw Hospital, Zhejiang University School of Medicine, Hangzhou, China; ^2^Key Laboratory of Microbial Technology and Bioinformatics of Zhejiang Province, Hangzhou, China; ^3^Department of Clinical Laboratory, The First Affiliated Hospital, Zhejiang University School of Medicine, Hangzhou, China; ^4^Department of Clinical Laboratory, Sir Run Run Shaw Hospital, Zhejiang University School of Medicine, Hangzhou, China; ^5^Department of Clinical Laboratory, Zhejiang Provincial People's Hospital, Hangzhou, China; ^6^Department of Clinical Laboratory, Affiliated Hangzhou First People’s Hospital, Zhejiang University School of Medicine, Hangzhou, China

**Keywords:** *Cryptococcus neoformans*, *Cryptococcus gattii*, antifungal susceptibility testing, multilocus sequence typing (MLST), mating type (MAT), genotype, whole-genome sequencing (WGS)

## Abstract

*Cryptococcus* spp. is a complex species that often causes cryptococcosis, which is one of the most common opportunistic infections in adults living with HIV and has very high morbidity and mortality rates. This study aimed to investigate the antifungal susceptibility profiles and epidemiological characteristics of the *Cryptococcus neoformans* species complex (CNSC) and the *Cryptococcus gattii* species complex (CGSC) in Zhejiang Province, China. A total of 177 CNSC and 3 CGSC isolates were collected, and antifungal susceptibility was tested by FUNGUS 3 and verified with an E-test. Moreover, multiple classification methods and genomic analyses were performed. The majority of the isolates (96.11%) were *C*. *neoformans* (formerly *C*. *neoformans* var. *grubii*) (ST5-VNI-A-α). Our study highlights that most of the patients with cryptococcosis were non-HIV patients in China, and nearly half of them did not have underlying diseases that led to immune insufficiency. Most of the *Cryptococcus* spp. isolates in this study were sensitive to common antifungal drugs. Two 5-flucytosine (5-FC)-resistant strains were identified, and *FUR1* mutation was detected in the 5-FC-resistant isolates. Typing based on whole-genome sequencing (WGS) showed better discrimination than that achieved with multilocus sequence typing (MLST) and indicated a clear population structure. A phylogenetic analysis based on WGS included more genomic information than traditional classification methods.

## Introduction

Cryptococcosis is primarily caused by infection with the *Cryptococcus neoformans* species complex (CNSC) or *Cryptococcus gattii* species complex (CGSC). CNSC has been classified as *C*. *neoformans* (serotype A, formerly *C*. *neoformans* var. *grubii*) which includes four genotypes: VNI, VNII, VNBI, and VNBII, *C*. *deneoformans* (serotype D, genotype VNIV, formerly *C*. *neoformans var*. *neoformans*) and *C*. *neoformans* × *C*. *deneoformans hybrid* (serotype AD, genotype VNIII). *C*. *gattii* was classified into distinct species, including *C*. *gattii* (genotype VGI, formerly *C*. *neoformans* var. *gattii*), *C*. *deuterogattii* (VGII), *C*. *bacillisporus* (VGIII), *C*. *decagattii* (VGIIIc/VGIV hybrid), *C*. *tetragattii* (VGIV) and other unnamed species (VGV), which can be classified into serotypes B or C (Hagen et al., 2015; Kwon-Chung et al., 2017; Montoya et al., 2021). *Cryptococcus* spp. often causes clinical invasive fungal diseases (IFDs), such as cryptococcal meningitis/meningoencephalitis (CM), pulmonary cryptococcosis and cryptococcal sepsis, and among these, CM is the most common and serious type of cryptococcosis. It is estimated that 223,100 cases of cryptococcal meningitis are reported worldwide every year (Rajasingham et al., 2017). Cryptococcosis is known as one of the most common opportunistic infections in adults living with HIV (Rajasingham et al., 2017), but it also occurs in non-HIV populations with underlying diseases, such as diabetes, chronic liver disease, kidney disease, lung disease, malignancies, long-term steroid therapy or solid organ transplants, that may affect the immune status (Xiaobo Feng et al., 2008; Liaw et al., 2010; Zhu et al., 2010; Williamson et al., 2017).

For the clinical treatment of cryptococcosis, particularly CM, the combination of amphotericin B (AMB) and 5-flucytosine (5-FC) followed by fluconazole (FLC) maintenance therapy is recommended (Perfect et al., 2010; Liu et al., 2018). Although research on antifungal drug resistance is important, there is no defined clinical breakpoint (CBP) for antifungal agents except AMB (resistant if MIC >1 mg/L). The epidemiological cut-off values (ECOFFs) indicated in the European Committee on Antimicrobial Susceptibility Testing (2022) guidelines for wild-type (WT) *C*. *neoformans* are defined as MIC ≤1 mg/L for AMB and MIC ≤0.5 mg/L for posaconazole and voriconazole, and no ECOFFs for other antifungal agents have been established. For *C*. *gattii*, the ECOFFs are 0.5 mg/L and 1 mg/L for AMB and posaconazole, respectively, no ECOFFs have been indicated for other antifungal agents, and no CBPs have been reported for any antifungal agents (European Committee on Antimicrobial Susceptibility Testing, 2022). It is very important to further study the mechanisms of resistance to these drugs because this resistance increases the failure rate of induction treatment (Perfect et al., 2010; May et al., 2016). Further studies are needed to aid the establishment of cryptococcal CBPs in the future.

Several molecular typing systems have been applied for *Cryptococcus* spp. The commonly used methods include genotype (Meyer et al., 2009), serotype (T G Mitchell JRP., 1995), and mating type (Chaturvedi et al., 2000) analyses. The genotype can be determined with many methods. In this study, we used two band-based methods, M13-based PCR fingerprinting (FP) and URA5-RFLP analysis, and two sequence-based methods, MLST and whole-genome sequencing (WGS). The serotype and mating type can be identified by target sequencing using PCR. MLST (Meyer et al., 2009) provides high discriminatory power, has good repeatability across different laboratories, and is inexpensive. Thus, MLST is a major and important method for strain typing in epidemiological studies of *Cryptococcus* spp. (Meyer et al., 2009). However, the MLST system (Meyer et al., 2009) is not suitable for the molecular typing of hybrids because it fails to amplify certain alleles (Li et al., 2012; Samarasinghe and Xu, 2018; Cogliati et al., 2020). To overcome this problem, new primers were designed to enable the typing of AD hybrids in 2020 (Cogliati et al., 2020).

Multilocus sequence typing was used to determine the internal 469 ~ 723-bp nucleotide sequence of seven housekeeping genes for further typing (Meyer et al., 2009). ST5/VNI is the major molecular type of CNSC in China. Its appearance suggests low genetic diversity because the current genotyping method covers few genes, but the genomes of *Cryptococcus neoformans* var. *neoformans JEC21* and *Cryptococcus gattii WM276* are 18.57 Mb and 18.4 Mb, respectively, and include 14 chromosomes (Loftus et al., 2005; D’Souza et al., 2011). Previous analysis showed that ST5 has different subtypes, which was observed following the detection of single nucleotide polymorphism (SNPs) variants (Zhou et al., 2022), and WGS can further be used to analyse resistance mechanisms and simultaneously acquire other typing features (Zang et al., 2022; Zhou et al., 2022). Considering the complexity of *Cryptococcus* spp. genomes and the development of genome sequencing technology, typing based on WGS has achieved great success in the discrimination of species (Firacative et al., 2016; Wongsuk et al., 2020) and may further reveal the evolutionary relationship and population structure of *Cryptococcus* spp. isolates.

In previous studies of *Cryptococcus* spp. in Zhejiang, 98.1% (51/52) of cryptococcemia isolates were identified as *C*. *neoformans* var. *grubii* ST5-VNI-α. Most patients (64.80–71.4%) with cryptococcosis were not infected with HIV, according to data from a general hospital in Zhejiang Province, which is also a designated HIV hospital (Fang et al., 2020; Zhao et al., 2021). To collect more strains to further and more comprehensively explore the clinical characteristics, drug susceptibility and molecular characteristics of *Cryptococcus* spp. in Zhejiang Province, we collected all preserved strains of clinically isolated *Cryptococcus* spp. in 4 hospitals in Zhejiang Province from 2012 to 2018, and antimicrobial susceptibility testing (AST), molecular typing, and genomic analysis were performed.

## Results

### Isolates and clinical information collection

In total, 180 nonrepetitive isolates were collected from four hospitals (99, 64, 14, and 3 from each hospital) in Zhejiang Province in China, and these included 177 CNSC isolates and three CGSC isolates. All 180 patients were diagnosed as exhibiting positive *Cryptococcus* spp. culture; among these, 68.33% (123/180), 16.67% (30/180) and 13.89% (25/180) were diagnosed with cryptococcal meningitis, pulmonary cryptococcosis and sepsis, respectively, and 1 patient diagnosed with soft tissue infection and 1 patient diagnosed with abdominal infection were also observed. Correspondingly, 123 strains, which included 3 isolates of CGSC, were isolated from the cerebrospinal fluid, and 30, 25, one and one strains were isolated from lung-related specimens, blood culture, skin-soft tissue, and ascites, respectively.

**Figure 1:**
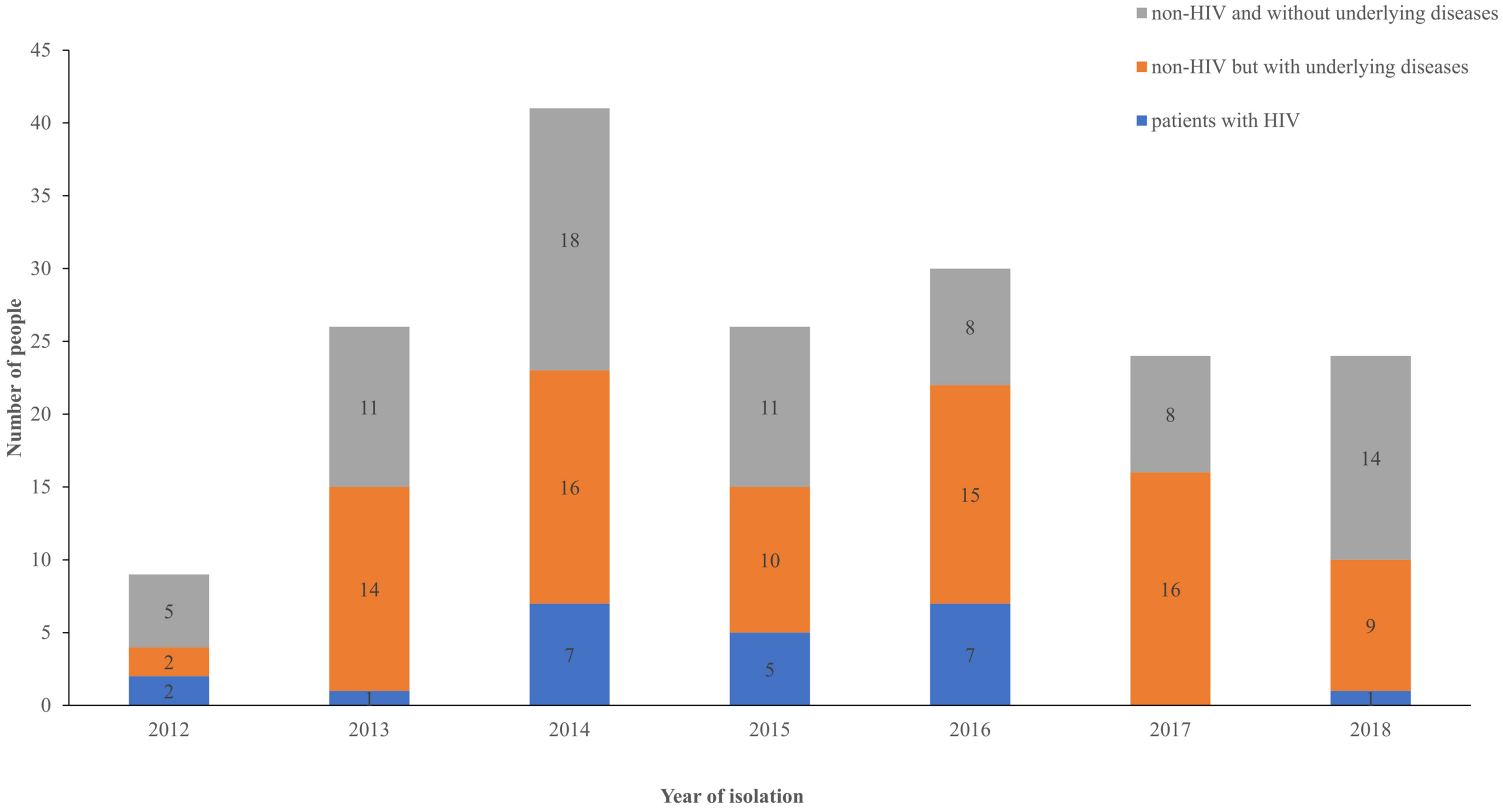
Distribution of patients among the three groups over time. Blue, HIV group; orange, non-HIV group with underlying diseases; gray, non-HIV group without underlying diseases.

The patients were classified into three groups based on immune status: HIV-associated (12.78%, 23/180), non-HIV but with underlying diseases that may influence the immune system (45.55%, 82/180), and non-HIV and without underlying diseases (41.67%, 75/180). The number of HIV-related cases of infection was lower than the number of cases observed in the other two groups, as shown in Figure 1. Pearson correlation analysis was performed to assess the increasing trend of each group over time. No statistical significance was observed in each group (*p* > 0.5).

### Molecular typing

Among 177 CNSC isolates, 173 were *C*. *neoformans* represented by the genotype VNI, serotype A, and MAT α. Another 4 isolates of CNSC were AD hybrids: RM007 was a hybrid of VNIII-AD, MAT α/a; ZY056 and SR017 were hybrids of VNIII-AD-α; and SR008 was also an AD-MAT α hybrid. Notably, the genotypes of SR008 that were observed using the two genotyping methods were inconsistent; VNIII was genotyped by M13-based PCR fingerprinting (FP), whereas the result of URA5-RFLP was VNIV. The genotyping electrophoresis diagrams of 4 AD hybrids and four reference strains are shown in Supplementary Figures S1A,B. Three isolates of CGSC exhibited the same mating type, MAT α, but different genotypes, including VGI (*n* = 1) and VGII (*n* = 2).

Through MLST typing, 173 isolates of *C*. *neoformans* were identified into 5 STs: ST5 was the predominant ST (*n* = 155), followed by ST31 (*n* = 8), ST79 (*n* = 5), ST81 (*n* = 3) and ST359 (*n* = 2). The CGSC isolates (*n* = 3) included three STs, namely, ST215 (RM009 VGI, from Taizhou), ST328 (ZY002 VGII, from Shaoxing) and the novel ST577 (SR06 1 VGII, from Taizhou). However, this approach failed to type the 4 AD hybrids even with the newly designed primers (Cogliati et al., 2020). Specifically, 12 ATs, including GPDVNIV 4, PLBVNIV 2, LACVNI 2, IGSVNI 1, IGSVNIV 2, and CAP59VNI 1, could not be amplified.

**Figure 2:**
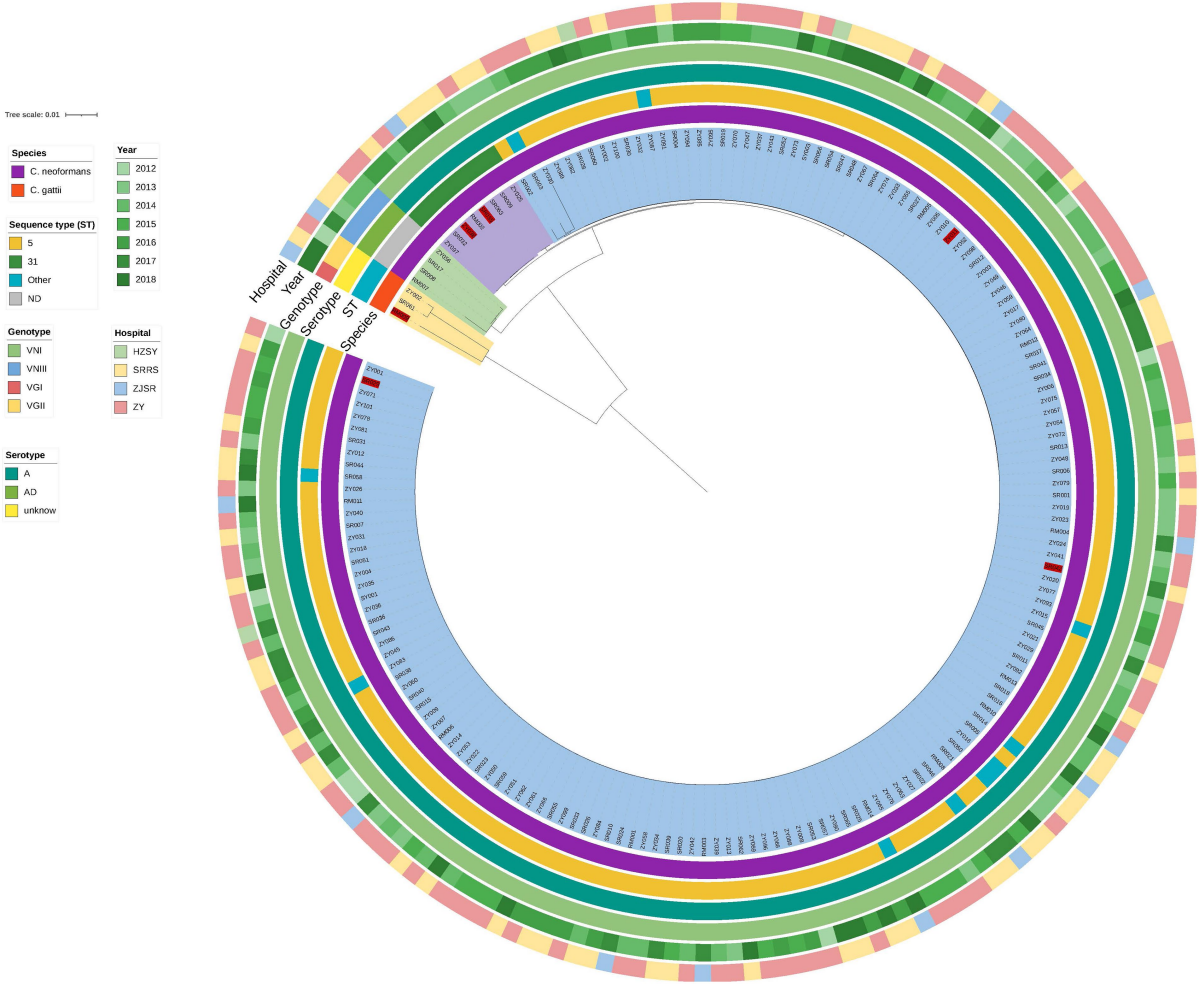
Phylogenetic tree of 180 isolates based on WGS. The innermost circle is the phylogenetic tree based on WGS. All strains were classified into two large clusters. The cluster indicated in yellow (corresponding to the bright orange of the “species” in the first circle) represents the CGSC*s*, and the other (corresponding to the dark purple of the “species”) represents *C*. *neoformans*. The red labelled strains represent strains with high MICs. The second, third, and fourth circles correspond to the sequence type (ST), genotype and serotype, respectively. The cluster of CGSC*s* contains three strains, RM009-ST215-VGI-α, SR061-ST552-VGII-α, and ZY002-ST328-VGII-α, and these molecular types were significantly different from *C*. *neoformans*. In the *C*. *neoformans* cluster, the strains were divided into three clusters (green, purple, and blue); the cluster indicated in green had four hybrids, RM007-VNIII-AαDa, ZY056, SR008, and SR017, which were all VNIII-AD-α; the cluster indicated in purple had 8 strains, all of which were ST31-VNI-A-α; the large cluster, which is represented in blue, had 165 strains, including ST5 (*n* = 155, the dominant ST), ST79 (*n* = 5), ST81 (*n* = 3), and ST359 (*n* = 2), all of which were VNI-A-MAT α. The isolation year (the fifth circle) and hospital (the sixth circle) showed a scattered distribution, and no significant difference in the corresponding strain species was detected.

The phylogenetic tree obtained based on WGS grouped the 180 isolates distinctly, which indicated that the CNSCs and CGSCs belonged to two major clusters (Figure 2). Furthermore, CNSCs were classified into three subclusters: the ST5 cluster, ST31 cluster and hybrid cluster. The ST5 cluster included 165 isolates with sequence types that included ST5, ST79, ST81, and ST359 was the dominant cluster with genotype VNI, serotype A, and MAT 𝛼 and was persistent during the time period in the study region, indicating its wide and continuous epidemic spread. The ST31 cluster contained 8 ST31 isolates. The phylogenetic tree grouped the AD hybrid isolates well, although MLST typing failed. The hybrid isolates were substantially different from those in the other two clusters. Among the hybrid clusters, three strains with the same mating type were closer to each other than to isolate RM007, which had a mating type of a/𝛼 (the other 179 strains in this study were all MAT 𝛼).

### Antimicrobial susceptibility testing and drug resistance mechanism

Only a few strains with high MICs were detected. The MIC range, geometric mean (GM), MIC50 and MIC90 of 5 common antifungal agents are shown in Table 1. Although no AMB-resistant strains were found, 6 isolates with high MICs (Table 2), 2 for 5-FC and 4 for azoles, were identified. Among isolates with high MICs for azoles, 1 isolate had high MICs for all 3 drugs and belonged to cluster 31 (FLC 16 mg/L, ITR 1.5 mg/L, and VRC 0.64 mg/L), 1 isolate had high MICs for FLC (16 mg/L) and ITR (1 mg/L) and belonged to cluster 5, and the other 2 isolates with high MICs (16 mg/L and 24 mg/L) for FLC belonged to clusters 5 and 31 (Figure 2). Two strains had high MICs for 5-FC, with MICs greater than 32 mg/L. RM009 is a type of *C*. *gattii* (ST215, VGI, MAT α), and ZY011 is a type of *C*. *neoformans* var. *grubii* (ST5, VNI, MAT α).

**Table 1:** Antifungal susceptibilities of five antifungal agents.

Antifungal agent	Range (μg/mL)	GM (μg/mL)	MIC50 (μg/mL)	MIC90 (μg/mL)
Amphotericin B	0.38–0.75	0.562	0.5	0.5
5-Flucytosine	0.064 – >32	3.574	4	4
Fluconazole	0.125–24	2.765	2	8
Voriconazole	0.016–0.64	0.124	0.13	0.5
Itraconazole	<0.125–1.5	0.192	0.13	0.25

GM, geometric mean of MICs.

**Table 2:** General information of 6 isolates with high MICs.

Identification number	Species	Year	ST	Genotype	MAT	5-FC	AMB	FCA	ITR	VRC
RM009	*C*. *gattii*	2018	ST215	VGI	α	>32	<0.5	2	0.25	0.125
ZY011	*C*. *neoformans*	2013	ST5	VNI	α	>32	<0.5	4	<0.125	<0.06
SR042	*C*. *neoformans*	2017	ST5	VNI	α	4	<0.5	24	0.5	0.047
ZY038	*C*. *neoformans*	2014	ST31	VNI	α	4	<0.5	16	0.5	0.5
SR029	*C*. *neoformans*	2016	ST5	VNI	α	4	<0.5	16	1	0.5
SR035	*C*. *neoformans*	2017	ST31	VNI	α	4	<0.5	16	1.5	0.64

**Figure 3:**
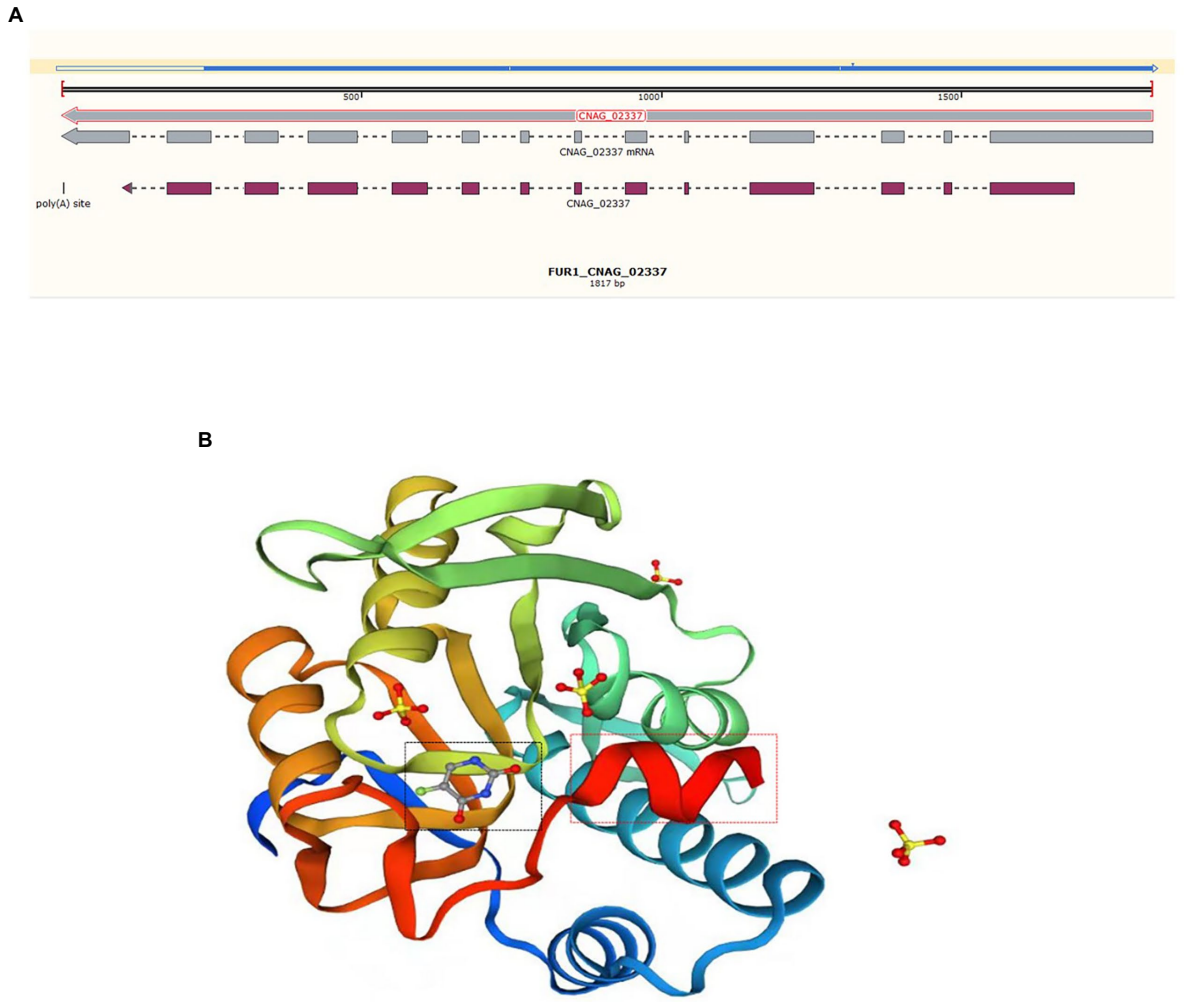
Sequence alignment and protein structure of FUR1. (**A)** DNA sequence alignment of the *FUR1* gene of ZY011 and the reference sequence (CNAG_02337). The blue line above refers to the sequence of ZY011, and the different sequences are illustrated with a hollow line. The gray line is representative of the reference sequence of the gene and mRNA, and the mauve line represents the exons. **(B)** We revealed the protein structure of *FUR1*, and the different sequences are highlighted with red rectangles. This region is near the 5-FU-binding site (black rectangle).

The potential drug resistance mechanism was explored. However, no mutation in the *ERG11* gene was observed in strains with high MICs for azoles after comparison with the sequence of the reference gene from H99 (*ERG11*, JQ044790, CNAG_00040). To determine the mechanism underlying 5-FC resistance, mutations in genes belonging to the *FCY2-FCY1-FUR1* pathway were detected. Regarding RM009, there were 5 synonymous mutations in the *FCY2* and *FCY3* genes, 4 synonymous mutations and 2 meaningful mutations (T54C (Ser-Gly) and G460A (Ser-Phe)) in the *FCY1* gene, and 3 synonymous mutations in the *FUR1* gene. Another strain, ZY011 (*C*. *neoformans*-ST5-VNI-A-α), exhibited no mutations in the *FCY2-4* and *FCY1* genes after comparison with the sequence of the reference gene from strain H99 (VNI, ST2, serotype A), but the sequence alignment of the *FUR1* gene was significantly different from the reference sequence, which lacked approximately 200 bp. This missing region started at the 206th amino acid (Figure 3A). The sequence integrity was verified by PCR and Sanger sequencing. The missing region is close to the 5-fluorouracil (5-FU) binding site and may influence the binding of 5-FU and mediate the increase in the MIC for 5-FC (Figure 3B).

## Discussion

As revealed by previous studies (Zhu et al., 2010; Fang et al., 2020; Fu et al., 2020; Zhou et al., 2020; Zhao et al., 2021), there are many more non-HIV patients in China than in other countries. In our study, we observed the same phenomenon: the proportion of patients with HIV was 12.78%. This finding may be due to the lower prevalence of HIV infection and the implementation of primary FLC prophylaxis in patients with CD4 counts <100 cells/μL in China (Organization WH, 2011; Parkes-Ratanshi et al., 2011; Oladele et al., 2017; Williamson et al., 2017). More importantly, this phenomenon indicates the importance of monitoring non-HIV-associated cryptococcosis even in apparently immunocompetent individuals (Williamson et al., 2017). In the United States, 20% of HIV-negative patients have no underlying conditions, such as a history of steroid therapy or malignant tumours (Williamson et al., 2017). Among non-HIV patients in this study, the presence of diseases affecting the immune system did not appear to increase the risk of *Cryptococcus* spp. infection since the proportions of patients with and without underlying diseases were nearly equal (45.55% vs. 41.67%). CNS-related infections were the main type of disease observed among HIV-negative patients (67.52%, 106/157). We reviewed the previous reports of Chinese researchers (Zhu et al., 2010) reported the clinical characteristics of 154 patients with cryptococcal meningitis over a 10-year period, the major of patients (66.9%) were otherwise apparently healthy and only 33.1% patients had predisposition factors (Zhu et al., 2010), there are also more reports from China that about 60% of cryptococcosis were from immunocompetent individuals, it is speculated that Chinese population might be more susceptible to cryptococcal infections than other ethnic groups (Fang et al., 2015).

In previous *Cryptococcus* spp. studies, many typing methods, such as RAPD, PCR fingerprinting, AFLP, MLMT, and MLST, have been employed and have mainly focused on genotyping (Hong et al., 2021). Due to their variable discrimination power, each of these methods has expanded knowledge regarding the genomic diversity of *Cryptococcus* spp. (Meyer et al., 2009; Hagen et al., 2015; Hong et al., 2021). We used band-based methods with the molecular types VNI–VNIV, VNB of CNSC and VGI-VGV of CGSC to assess this type of genomic diversity. With the development of sequencing technology, sequence base typing methods have provided more discriminatory power, repeatability and comparability. Thus, MLST and WGS are becoming increasingly popular. Using the MLST system, we identified lineages using sequence types. However, because MLST only includes the analysis of seven housekeeping genes, MLST may provide unilateral results, unlike WGS typing. Genome analysis in eukaryotes, including assembly, annotation, SNP calling, and phylogenetic tree reconstruction, is extremely difficult and requires expert bioinformatic knowledge and high-performance computers (Desjardins et al., 2017). In our study, we proved that even with roughly processed genomes, the results presented real genomic diversity and were clearly distinguished at the species level and lineage level. Using genome assemblies, Mashtree was used to reconstruct the phylogenetic tree efficiently. Not only can CNSCs and CGSCs be clearly distinguished, but subclusters in the major cluster can also be classified, as reported by Ziyi Zhou et al. in 2022 in a study that showed that ST5 has different subtypes based on the detection of single nucleotide polymorphism (SNPs) variants (Zhou et al., 2022), and the ST5 cluster and ST31 cluster can be roughly considered CC5 and CC31 (Fan et al., 2016; Cogliati et al., 2019; Thanh et al., 2019). Furthermore, the relationship between AD hybrids and other CCs could be clearly distinguished. These results improved the typing methods and revealed the biodiversity of *Cryptococcus* spp. in China (Zang et al., 2022; Zhou et al., 2022).

The latest research has shown that the nonsusceptibility rate (intermediate and resistant) of *Cryptococcus* spp. with FLC reached 25.9% (Simwami et al., 2011), which was significantly higher than the value reported 9 years ago (9.5%) (Wang et al., 2012; Xiao et al., 2018). This finding raises concerns about the increased drug resistance rate. Mutation of the *ERG11* gene is the most common mechanism underlying azole resistance. It has been reported that the G484S, Y145F and G470R mutations of *ERG11* are related to azole resistance (Rodero et al., 2003; Sionov et al., 2012; Gago et al., 2017; Zhang et al., 2019). However, no mutation was found in *ERG11* in these high-MIC strains in our study.

In particular, two strains with high MICs of 5-FC (>32 mg/L) were found in this study: 1 strain of *C*. *gattii* and 1 strain of *C*. *neoformans*. To date, the drug resistance rate of 5-FC with *Cryptococcus* spp. is 1–25% (Cuenca-Estrella, 2001; Govender et al., 2011; Espinel-Ingroff et al., 2012; Chang et al., 2021). The metabolic pathway of *FCY2-FCY1-FUR1* is related to 5-FC biochemical reactions and may cause 5-FC resistance due to its mutations. The heterozygous G/T mutation at position 145 of the *FCY2* gene and the nonsense mutation C505T have been reported. In addition, the T26C point mutation of the *FCY1* gene leads to a change in the protein (M9T), which can lead to cross-resistance to 5-FC and FLC (Papon et al., 2007; Florent et al., 2009). Considering the mutations identified in the *C*. *gattii* isolate RM009, we assumed that the two nonsynonymous mutations in the *FCY1* gene may be responsible for 5-FC resistance, but this finding needs further research. There were substantial differences regarding the *C*. *neoformans* isolate (ZY011); no exon mutation was found in the *FCY2-4* and *FCY1* genes, but the sequence of the *FUR1* gene was significantly different from the reference sequence. The identical sequence ended in an exon sequence, and because there were no other ways to identify the sequence of mRNA or protein, we could not predict the actual change in this gene. Considering that the changed region is near the 5-FU-binding site, mutations in the *FUR1* gene may cause the binding of pyrimidines to be blocked or inefficient, which may be the main factor underlying resistance.

The MICs of the 5 anti-cryptococcal drugs in this study were generally not high, there was no amphotericin B-resistant strain, most of the *Cryptococcus* spp. strains were sensitive to azoles, the MICs of several NWTs were generally not high, only two 5-FC-resistant strains were identified, these provides a more detailed reference for the empirical therapy of cryptococcosis, and our study supplements the available information regarding cryptococcal infections in Zhejiang Province for the global fungal database. These findings prove that simply processed genome sequences also provide convincing evidence of lineages.

## Conclusion

In mainland China, the non-HIV population is the most commonly susceptible to cryptococcosis, and *Cryptococcus neoformans* (ST5-VNI-A-α) is the predominant pathogen. The isolated hybrids and CGSCs were rare; of particular interest, three strains of *C*. *gattii*, which are rare in the clinic, all caused meningitis and occurred in younger patients without underlying diseases. Phylogenetic analysis based on WGS is not band-based, covers more genomic information and therefore shows higher discriminatory power than traditional typing methods. Anti-cryptococcal drug-resistant isolates were rare, but isolates with high MICs were identified. Mutations in *FCY2-FCY1-FUR1* were detected in the 5-FC-resistant isolate, and this finding deserves further study.

## Limitations

However, there are also some limitations in this study. First, data may be outdated or incomplete because many data were collected retrospectively through electronic medical records. Second, the proportion of HIV in underlying diseases would be biased in that only one of the four hospitals in the study is a designated hospital for HIV treatment. Third, due to the limitation of diagnosis methods and the lack of understanding of cryptococcosis in early years, not all *Cryptococcus* spp. strains in four hospitals were collected at the same time, resulting in sample bias. A better epidemiological investigation design is needed in the future.

## Materials and methods

### Ethics statement

The study was approved by the ethics review board (found in Supplementary material).

### Isolate collection

Isolates of CNSC and CGSC from four hospitals were collected between 2012 and 2018. All isolates were identified by matrix-assisted laser desorption/ionization time-of-flight mass spectrometry (MALDI-TOF MS). Clinical information, such as age, sex and underlying diseases, was collected by reviewing the patients’ electronic medical records. Based on this information, all cases were classified into three groups: (Kwon-Chung et al., 2017) HIV-associated (Hagen et al., 2015) non-HIV but with underlying diseases that may influence the immune status, and (Montoya et al., 2021) non-HIV and without underlying diseases. The following circumstances were considered underlying diseases: receipt of solid organ transplantation, connective tissue disease, aggressive cancer treatment, use of immunosuppressants or glucocorticoids, malignancies, haematologic malignancies, cirrhosis, diabetes mellitus, and uraemia (Zhu et al., 2010; Williamson et al., 2017; Beardsley et al., 2019). The risk level of HBV reactivation is moderate or high when treat with medium (10–20 mg/day) or high dose (≥20 mg/day) prednisone for ≥4 weeks, medium (10–20 mg/day) or high dose (≥20 mg/day) for ≥4 weeks (Lau et al., 2021), and prednisone therapy ≥7.5 mg/day were associated with a higher herpes zoster risk (Pappas et al., 2015). In the present study, patients receiving continuous glucocorticoids therapy that due to connective tissue diseases were included in the glucocorticoid group (excluding glucocorticoid containing chemotherapy); All patients with diabetes were type 2 diabetes. The “non-HIV but without underlying disease” group included patients who had no disease or some diseases that were well controlled, such as gout, hypertension, chronic hepatitis B, chronic kidney disease, and anaemia.

Further statistical analysis was performed, and the results are expressed as the means ± standard deviations.

### Reference strains

Eight reference strains were used as controls: WM148 (VNI, serotype A, MATα), WM626 (VNII, serotype A, MATα), WM628 (VNIII, serotype AD, MATα/a), WM629 (VNIV, serotype D, MATα), WM179 (VGI, serotype B, MATα), WM178 (VGII, serotype B, MATα), WM161 (VGIII, serotype B, MATα) and WM779 (VGIV, serotype C, MATα) (Meyer et al., 2009).

### DNA extraction

Genomic DNA was extracted from the collected isolates with ZR Fungal/Bacterial DNA Kits (catalogue no. D6005, The Epigenetics Company) according to the manufacturer’s recommendations. The extracted DNA was used for MLST, genotype, serotype and next-generation sequencing analyses.

### Molecular typing

The genotypes were verified using two methods, M13-based PCR fingerprinting (FP) and URA5-RFLP analysis, according to previously described protocols (Meyer et al., 2009). The serotype and mating type were determined by PCR, and the six related primer pairs of *C*. *neoformans* were used to determine the strain serotype and mating type according to previously described protocols (Chaturvedi et al., 2000; Dou et al., 2015; Wu et al., 2015).

### Genomic analysis

Next,-generation sequencing of the strains was performed on the Illumina HiSeq X Ten platform. The raw genomic sequence data passed a quality control assessment, as demonstrated using fastqc (Andrews, 2010) and multiqc (Ewels et al., 2016). Genomes were then assembled using Shovill (Seemann et al., 2019, unpublished)^1^ with an average depth of 77.1. Utilizing the whole-genome sequence, a mash distance-based phylogenetic analysis was performed with Mashtree (Katz et al., 2019), and the results were visualized using the iTOL web service.^2^

ST type was determined by screening the genome data from each isolate on Cryptococcus spp. MLST database.^3^ The alleles that could not be matched were verified using the consensus ISHAM protocol by PCR and by sequencing seven loci: *CAP59*, *IGS1*, *GPD1*, *LAC1*, *PLB1*, *SOD1*, and *URA5* (Meyer et al., 2009; Hagen et al., 2015; Cogliati et al., 2020).

### *In vitro* antimicrobial susceptibility testing

The AST of 5 common antifungal agents, including amphotericin B (AMB), 5-flucytosine (5-FC), fluconazole (FLC), itraconazole (ITR), and voriconazole (VRC), was performed using an ATB FUNGUS 3 kit (bioMérieux, France). If the MICs were abnormally high or exhibited strong trailing growth, they were then verified using an E-test (bioMérieux). The preliminary determination of drug sensitivity was based on the Fungus 3 kit instructions and the CBPs of *Candida* spp. recommended by CLSI/NCCLS and the literature (CLSI, 2008; Tewari et al., 2012; Bongomin et al., 2018; Breakpoint Tables for Interpretation of MICs for Antifungal Agents, 2020). Quality control was performed using *Candida parapsilosis* (ATCC 22019) and *Candida krusei* (ATCC 6258) (Torres-Rodriguez and Alvarado-Ramirez, 2007; Performance Standards for Antifungal Susceptibility of Testing Yeasts, 2018).

To explore the possible drug resistance mechanism, the *ERG11* sequences of the high-MIC strains were compared with the reference sequences (*ERG11*, JQ44790, CNAG_00040) using BLAST.^4^ The changes in the metabolic pathway of *FCY2-FCY1-FUR1* were responsible for 5-FC resistance. Among *C*. *neoformans* isolates, pathway-related genes were compared with the reference gene of strain H99 (*FCY1* CNAG_00613, *FCY2* CNAG_01681, *FCY3* CNAG_04982, *FCY4* CNAG_04276, *FUR1* CNAG_02337). For *C*. *gattii* isolates, we used E566 (VGI, serotype B) as the reference genome. The missing sequence of the *FUR1* gene was verified by PCR and Sanger sequencing with the primers F-TGGATGAGATCATATGCCTG and R-GATTGCTGATTGGAAGGAC.

## Data availability statement

The datasets presented in this study can be found in online repositories. The names of the repository/repositories and accession number(s) can be found in the article/Supplementary material.

## Author contributions

YY, YJ, and ZZ: conceptualization. JZ, QY, YF, FZ, XL, and LF: data curation. JZ and ZZ: formal analysis. YY: funding acquisition. JZ, ZW, and LF: investigation. YC, YJ, and YY: methodology. JZ, ZW, and YY: project administration. ZZ, QY, YF, FZ, QC, and LF: resources. ZW, YC, XL, and YJ: software. YJ: supervision. JZ and ZW: validation and visualization. JZ: writing—original draft. ZW, YJ, and YY: writing—review and editing. All authors contributed to the article and approved the submitted version.

## Funding

This research was funded by the clinical research project of Zhejiang Provincial Department of Health (grant number 2018ZD030) and General Research Plan for Medical and Health of Zhejiang Province (Class A) (grant number 2020KY604).

## Conflict of interest

The authors declare that the research was conducted in the absence of any commercial or financial relationships that could be construed as a potential conflict of interest.

## Publisher’s note

All claims expressed in this article are solely those of the authors and do not necessarily represent those of their affiliated organizations, or those of the publisher, the editors and the reviewers. Any product that may be evaluated in this article, or claim that may be made by its manufacturer, is not guaranteed or endorsed by the publisher.

## Footnotes

^1^Seemann, T., Edwards, R., Da Silva, A., and Kiil, K. Shovill. (2019) (unpublished).

^2^ https://itol.embl.de/

^3^ www.mycologylab.net

^4^ https://blast.ncbi.nlm.nih.gov/Blast.cgi
