# Supplementary material for: Antifungal susceptibility and molecular characteristics of Cryptococcus spp. based on whole-genome sequencing in Zhejiang Province, China
Source: Front Microbiol. 2022 Nov 17;13:991703. doi: 10.3389/fmicb.2022.991703 (PMC9712201; doi:10.3389/fmicb.2022.991703)
Supplement: Supplementary file 2 [file Table_2.DOCX]

Supplementary Material

# Supplementary Figures and Tables

## Supplementary Figures


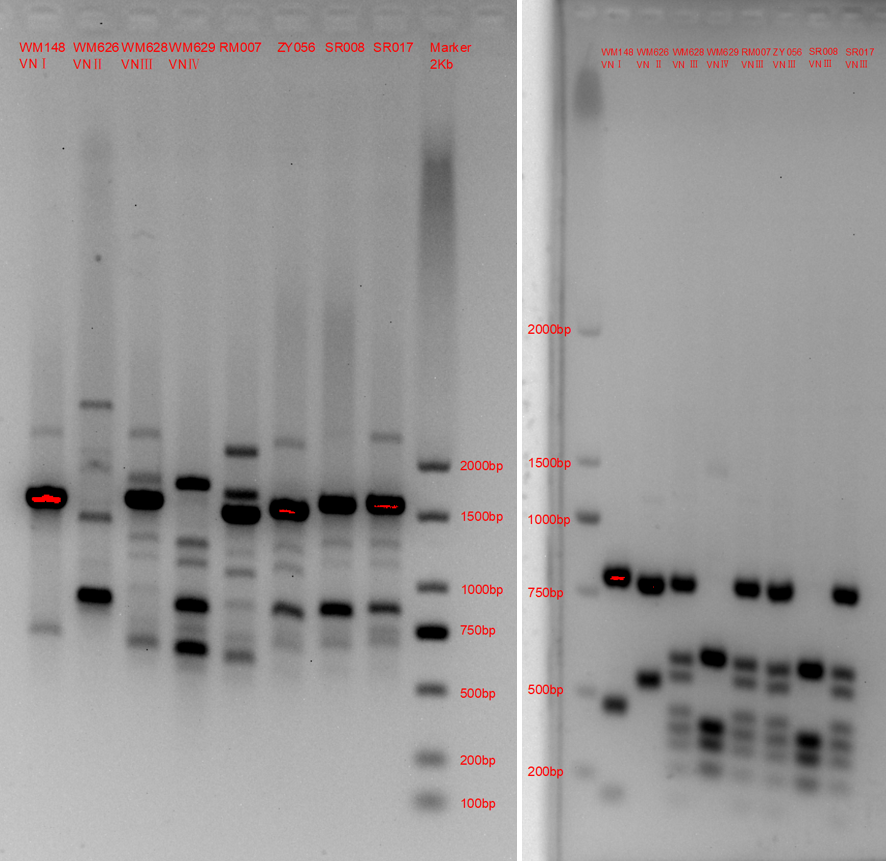


Supplementary Figure 1. The genotypes by M13-based PCR fingerprinting and URA5-RFLP analysis
Supplementary Figure 1a (left): M13-based PCR fingerprinting genotype using 1% agar gel, 0.5xTAE buffer, electrophoresis condition 110 V, 60 min.
Supplementary Figure 1b (right): URA5-RFLP analysis, using 1.8% agar gel, 0.5xTAE buffer, electrophoresis condition 110 V, 120 min.
